# Assessment of soil erosion in the Dongting Lake Basin, China: Patterns, drivers, and implications

**DOI:** 10.1371/journal.pone.0261842

**Published:** 2021-12-31

**Authors:** Jianyong Xiao, Binggeng Xie, Kaichun Zhou, Shana Shi, Junhan Li, Mingxia Yang, ChangChang Liu

**Affiliations:** School of Geographic Sciences, Hunan Normal University, Changsha, China; Northeastern University (Shenyang China), CHINA

## Abstract

Soil loss caused by erosion is a global problem. Therefore, the assessment of soil erosion and the its driving mechanism are of great significance to soil conservation. However, soil erosion is affected by both climate change and human activities, which have not been quantified, and few researchers studied the differences in the driving mechanisms of soil erosion depending on the land use type. Therefore, the spatiotemporal characteristics and changing trends of soil erosion in the Dongting Lake Basin were analyzed in this study. Geographic detectors were used to identify the dominant factors affecting soil erosion in different land use types. In this study, a sensitivity experiment was conducted to clarify the relative contributions of climate change and human activities to soil erosion changes. In addition, we studied the effects of different land use types and vegetation cover restoration on soil erosion. The results show that soil erosion in the Dongting Lake Basin decreased from 2000 to 2018. Human activities represented by land use types and vegetation coverage significantly contributed to the alleviation of soil erosion in the Dongting Lake Basin, whereas climate change represented by rainfall slightly aggravated soil erosion in the study area. The restoration of grassland vegetation and transfer of cultivated land to woodlands in the study area improved the soil erosion. The slope steepness is the key factor affecting the intensity of soil erosion in dry land, paddy fields, and unused land, whereas the vegetation coverage is the key factor affecting the intensity of soil erosion in woodland, garden land, and grassland. Detailed spatiotemporally mapping of soil erosion was used to determine the connections between soil erosion and potential drivers, which have important implications for vegetation restoration and the optimization of land use planning.

## Introduction

Soil erosion is one of the most serious global environmental problems, because it not only causes soil nutrient loss and land degradation, but also leads to many secondary environmental problems such as flooding, river siltation, and water pollution, which may affect the ecological security and sustainable development of the region [1, 2]. The occurrence of soil erosion is affected by multiple factors such as the topography, soil, land use, vegetation, and climate [3]. Climate change and human activities are the key factors driving the changes in soil erosion. The land use and vegetation cover are the most direct manifestations of human activities on the land surface. Rainfall directly affects soil erosion [4, 5]. Therefore, it is necessary to explore the dynamic characteristics of soil erosion, process of land use development, and ecological restoration to maintain ecosystem stability and provide long-term ecosystem services [6].

Many quantitative assessments of soil erosion have been carried out globally. An effective quantitative method is the establishment of soil erosion models. The most commonly used models are the Universal Soil Loss Equation (USLE) and Revised Universal Soil Loss Equation (RUSLE) proposed by the United States Department of Agriculture (USDA) [7, 8]. The RUSLE is based on computer and geospatial technology, and can be used to explore soil erosion and its spatial distribution with a reasonable accuracy at a limited cost [9, 10]. It has been widely used to quantitatively assess soil erosion at the regional and watershed scales [11–13]. Kijowska (2018) used a combination of the RUSLE and travel time concept and assessed the erosion in a basin [14]. Many scholars analyzed the spatiotemporal evolution of soil erosion by using trend analysis and the gravity center model based on detailed spatiotemporal mapping [15, 16]. Many soil erosion studies have been conducted with the aim to study certain plots or basins in China’s mountainous and hilly areas. The results of these studies have proven that the RUSLE is reliable and can be used for quantitative assessments of soil erosion in mountainous and hilly areas [17–19].

Soil erosion is a complex process that is affected by natural factors and human activities, such as rainfall, terrain conditions, soil characteristics, vegetation characteristics, and land use and management [3]. Rainfall characteristics determine the effects of raindrops and runoff on erosion, whereas the soil characteristics and topographical conditions affect the separability and mobility of soil particles [20, 21]. Vegetation can protect the soil surface from water erosion by intercepting rainfall, increasing infiltration, stabilizing soil aggregates, and reducing the soil erodibility [22, 23]. The effect of the climate on soil erosion is more complex. In addition to the direct influence of rainfall, climate also indirectly affects the soil conservation by controlling the vegetation growth [24]. Land use activities, including deforestation, overgrazing, farming, and inappropriate agricultural practices, are the main reasons for the deterioration of soil erosion. In contrast, soil and water conservation measures, such as slope reinforcement and bare land hardening can slow down soil erosion [25, 26]. Previously, the driving mechanism of soil erosion has been analyzed based on the topography [27]. Yu et al. suggested that climate change, especially precipitation, has a strong influence on soil erosion in the Loess Plateau [28]. Wei et al. analyzed a small watershed in the Loess Plateau and considered the Grain for Green Project to be the main reason for the decrease in the soil erosion in the Fangta watershed [29]. Although scholars have studied the effects of various factors, including climate, terrain, soil, and vegetation, on soil erosion [30–32], few have discussed their different effects on soil erosion depending on the land use type. Quantifying the effects of different factors on soil erosion for each land use type is conducive to the efficient implementation of soil and water conservation measures.

The Dongting Lake Basin is an important lake basin in the middle and lower reaches of the Yangtze River in China. Concentrated rainstorms caused by seasonal rainfall in mountainous and hilly areas have led to severe water erosion in the area. Because of intensive farming in the end of the last century, the Dongting Lake Basin has suffered from great soil and water loss [33]. Therefore, China implemented projects such as “returning farmland to forests” and “natural forest protection” to increase the vegetation cover and reduce soil erosion in 1999 [34, 35]. Moreover, rapid economic growth and human-land conflicts caused by urbanization stimulated profound changes in the spatial pattern of land use in the basin [36]. Although the focus of several studies was placed on small-scale basins and changes in soil erosion over a certain period [37–39], it is remains unknown how changes in rainfall, vegetation cover, and land use may affect the soil erosion in the Dongting Lake Basin, particularly under the influence of climate change and human activities.

The aim of this study was to explore the characteristics and driving mechanisms of soil erosion in the Dongting Lake Basin. The objectives were to: (1) reveal the spatiotemporal characteristics and changes of soil erosion of different land use types in the Dongting Lake Basin from 2000 to 2018; (2) identify the dominant factors driving soil erosion in different land use types; (3) clarify the relative contributions of climate change and human activities to soil erosion changes; and (4) explore the effects of different land use types transfer and vegetation cover restoration on soil erosion. The present results facilitate the adaptation of sustainable land management and soil conservation policies in areas similar to the Dongting Lake Basin.

## Materials and methods

### Study area

**Fig 1:**
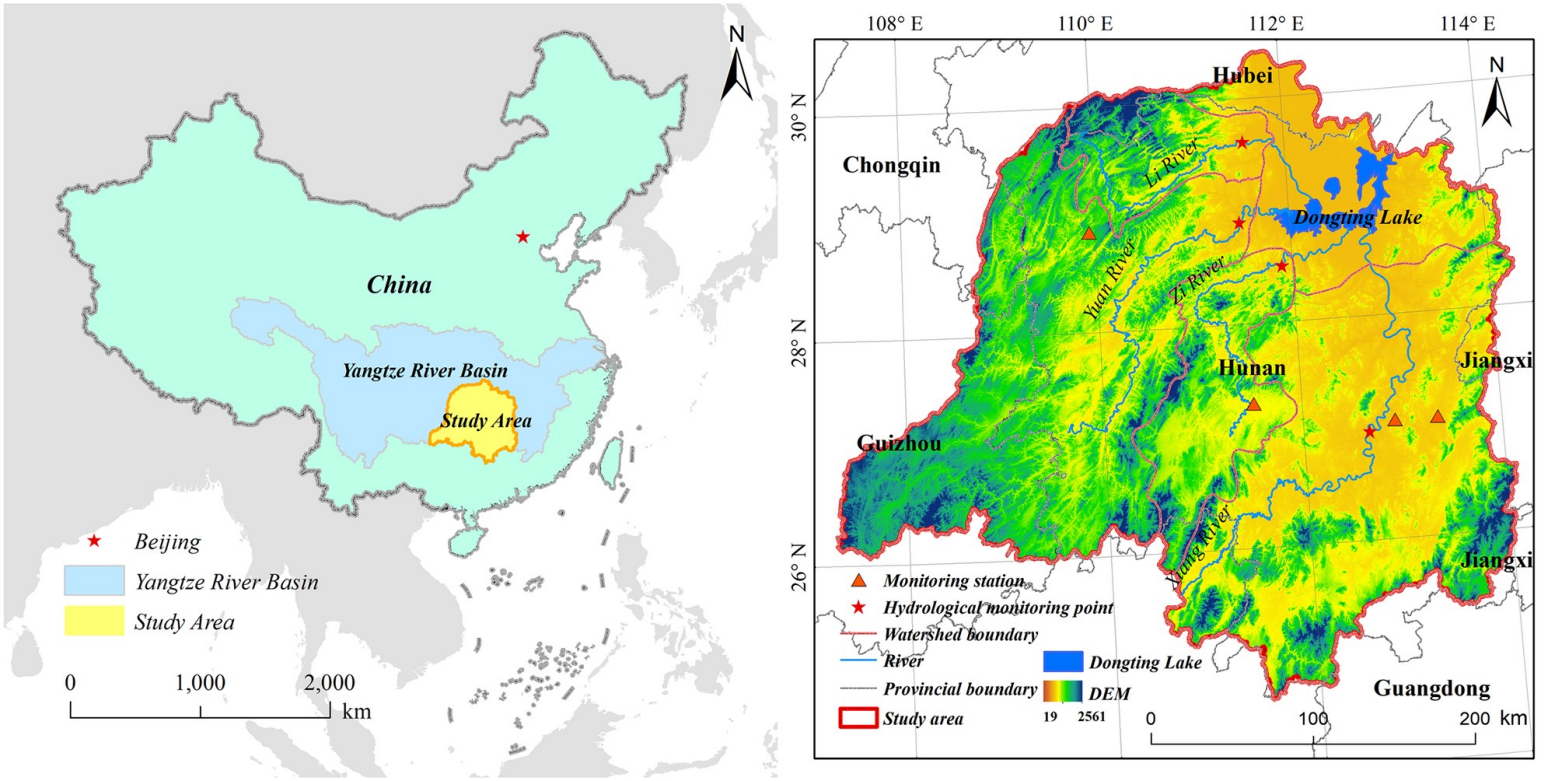
Location and topography of the study area. The image was created using ARCGIS 10.2 software with the authors’ data.

The Dongting Lake Basin is south of the middle reaches of the Yangtze River in China, between 24°38’–30°26’N and 107°16’–114°17’E, and covers a total area of 2628 hm^2^ (Fig 1). The elevation ranges from 19 to 2563 m above sea level. It straddles the second and third steps of China’s topography and is an important part of the Yangtze River Basin. This area has a superior geographical location with a complex topography, developed water system, and rich biological resources [40]. It includes the administrative regions of Hunan, Guizhou, Hubei, Guangxi Province, and Chongqing City. The Dongting Lake Basin has a horseshoe shape, with an opening on the top. Plains, basins, hills, mountains, rivers, and lakes characterize this area, which is surrounded by mountains are surrounding it on three sides. The basin lies in a humid subtropical monsoon climate zone, with an annual average temperature of 16.9°C and rainfall of 1400 mm/yr based on statistics collected from 2000 to 2018. It contains a river system with Dongting Lake as the center, Xiang river flowing in from the south, and the Zi, Yuan, and Li River flowing from the southwest and west.

#### Data sources

In this study data on the land use, Normalized Difference Vegetation Index (NDVI), Digital Elevation Model (DEM), rainfall, temperature, and soil were collected (Table 1). Kriging’s approach for spatial interpolation was applied to process the meteorological data from 52 meteorological stations in the study area and surrounding regions, and mean values were used to process MODIS data MODI13Q1 from the United States Geological Survey (USGS). To gather available data on paddy fields, dry land, woodland, garden land, grassland, the water area, construction land, and unused land, the initial land use data were reclassified. In addition, the Albers equal-area conic projection was used as geographic coordinate system, and the raster data were resampled to a resolution of 30 m.

**Table 1:** Basic data.

Data	Period	Resolution	Source	URLs
**Land use**	2000/01-2018/12	30 m	Chinese Academy of Sciences Resource and Environment Science Data Center	https://www.resdc.cn/
**NDVI**		250 m	United States Geological Survey	https://www.usgs.gov/
**Temperature**		Point	China Meteorological Data Net	http://data.cma.cn/
**Rainfall**				
**DEM**	present	30 m	Geospatial data cloud	http://www.gscloud.cn/
**Soil**		1000 m	Harmonized World Soil Database	https://iiasa.ac.at/
**Basin boundaries**		-	National Earth System Science Data Center	http://www.geodata.cn/
**Monitored data**		Point	Hunan Bulletin of Soil and Water Conservation	http://slt.hunan.gov.cn/hnsw/

### Methods

#### The RUSLE model

In this study, the RUSLE, which is a relatively mature model, was used to quantitatively evaluate soil erosion in the study area [41, 42]. The equation for the model is as follows:

A=R⋅K⋅L⋅S⋅C⋅P (1)

where *A* is the average annual soil erosion rate, also known as the annual average soil loss (t∙hm^-2^ a^-1^); *R* is the rainfall erosivity factor (MJ mm hm^-2^ h^-1^ a^-1^); *K* is the soil erodibility factor (MJ mm hm^-2^ h^-1^ a^-1^); *LS* is the slope length and steepness factor; *C* is the cover management factor; and *P* is the support practice factor. The factors *LS*, *C*, and *P* are dimensionless.

#### Linear regression analysis

Changes in the soil erosion were analyzed using linear regression, that is, based on the slope *k* of the least-squares linear regression equation [18]:

$$k=\frac{n\sum_{i=1}^{n}\left(i\times M_{A,i}\right)-\sum_{i=1}^{n}i\times \sum_{i=1}^{n}M_{A,i}}{n\times \sum_{i=1}^{n}i^{2}-{\left(\sum_{i=1}^{n}i\right)}^{2}}$$ (2)

where *k* is the slope of the soil erosion rate (*A*), which is directly proportional to the soil erosion trend. When *k* > 0, the soil erosion increases, and vice versa. In addition, the value of *n* is 5, *i* is the serial number of the year, and *M*_*A*_, *i* is the *A* value of the *i-*th year. A unified standard for the identification of *k* has not been established. Based on previous studies, the *k* value follows a normal distribution and can be divided into five grades [43]: substantial decrease (*k* ≤ –0.01), slight decrease (–0.01 ≤ *k* < –0.05), stability (–0.05 ≤ *k* < 0.05), slight increase (0.05 ≤ *k* < 0.01) and substantial increase (*k* > 0.01).

#### Geographical detector

A geographical detector tests the spatial stratified heterogeneity of a variable *Y* (i.e., the phenomenon that *Y* is more similar within strata than between strata, such as climate zones and many ecological variables) or the correlation between two variables *Y* and *X* according to the consistency of their spatial distributions [44]. The principle of the geographical detector is that variable *Y* is associated with variable *X* if their spatial distributions are identical. The correlation between *Y* and *X* can be determined as follows:

$${\text{P}}_{\text{X},\text{Y}}=1-\frac{1}{n\sigma^{2}}\sum_{i=1}^{m}X_{i},\sigma_{i}^{2}$$ (3)

where *P* is the detection index of the soil erosion rate (*A*), *Y* is the dependent variable (i.e., A in this study), *X* is the independent variable (i.e., Elevation, Slope, Soil, Rainfall, Temperature, NDVI), σ^2^ is the raster variance of *Y*, *n* is the total number of raster, *m* is the number of samples in the entire area, *i* is the number of secondary regions, and σ^2^_*i*_ is the secondary variance. Given that *P* ≠ 0, the *P* range of the model was set to [0, 1] and *P* = 0, which indicates that the *A* distribution is randomly distributed. A large *P* value indicates significant effects of the partitioning factors on *A*.

#### Sensitivity analysis

In this study, the relative contributions of climate change and human activities to soil erosion changes were analyzed using sensitivity analysis. In the sensitivity experiment, climate change was replaced with rainfall, and land use and vegetation cover were replaced by human activities. The following four numerical experiments were designed to identify the contribution of changes in rainfall, land use and vegetation coverage (NDVI) to soil erosion. The four numerical experiments included one control experiment (*Mo_Ctr*) and three sensitivity experiments (rainfall, land use type, and NDVI, which were denoted as *MO_R*, *MO_LU*, *MO_N*). First, the year 2000 was selected as the reference year for the sensitivity analysis. In the four numerical tests, *Mo_Ctr* utilized all actual data for the simulation. In the three sensitivity tests, the target parameters of the whole sequence were replaced with the data of the base year, and other data were used with real data. Thus, the contribution of one of the parameters to the target variable is

$$C_{i}^{k}=V_{Mo\_Ctr}^{k}-V_{i}^{k}$$ (4)

where *$C_{i}^{k}$* is the contribution of the *i-th* variable, *k* is the target variable (soil erosion rate), $V_{Mo\_Ctr}^{k}$ is the control experimental value for the *i-th* variable, and $V_{i}^{k}$ is the control experiment value for the *i-th* variable. Sensitivity experimental value of the *i-th* variable [45, 46].

#### Research framework

**Fig 2:**
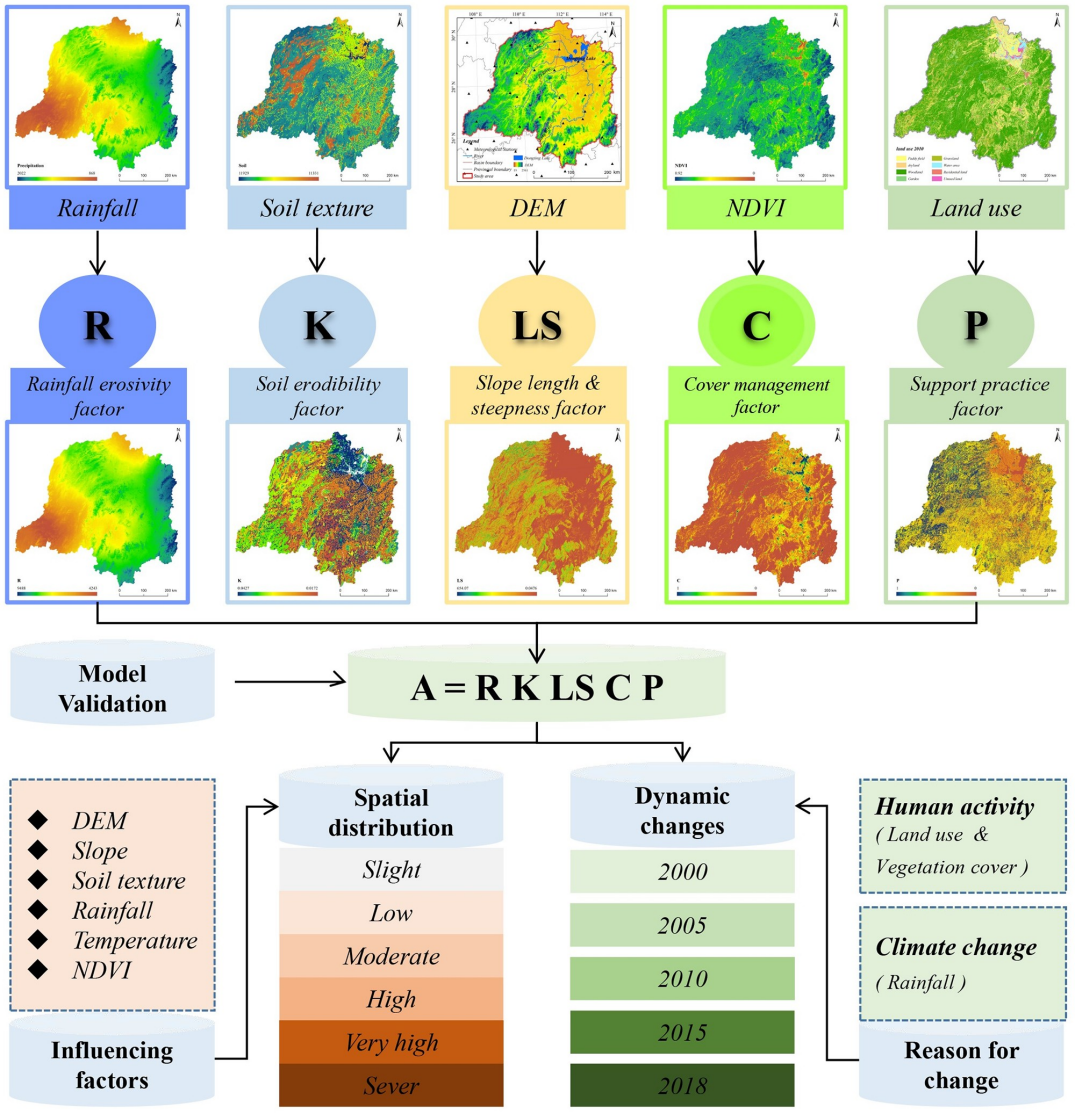
Flowchart of the spatiotemporal evolution of soil erosion and its influencing factors. The image was created using ARCGIS 10.2 software with the authors’ data.

In this study, the average soil loss in tons per hectare per year (A) was estimated with the RUSLE (Fig 2). The results were validated using actual measurements. The soil erosion was further classified into six levels according to A at the pixel level, that is slight (< = 5 t∙hm^-2^ a^-1^), low (5–25 t∙hm^-2^ a^-1^), moderate (25–50 t∙hm^-2^ a^-1^), high (50–80 t∙hm^-2^ a^-1^), very high (80–150 t∙hm^-2^ a^-1^), and severe (>150 t∙hm^-2^ a^-1^), following the Technological Standard of Soil and Water Conservation (SL190–2007, issued by the Ministry of Water Resources of China). To determine the variation in the soil loss along the slope gradient, the slope steepness was classified into six levels, i.e., < = 2°, 2°–6°, 8°–15°, 15°–25°, 25°–35°, and >35°.

## Results

### Spatial distribution of soil erosion

**Fig 3:**
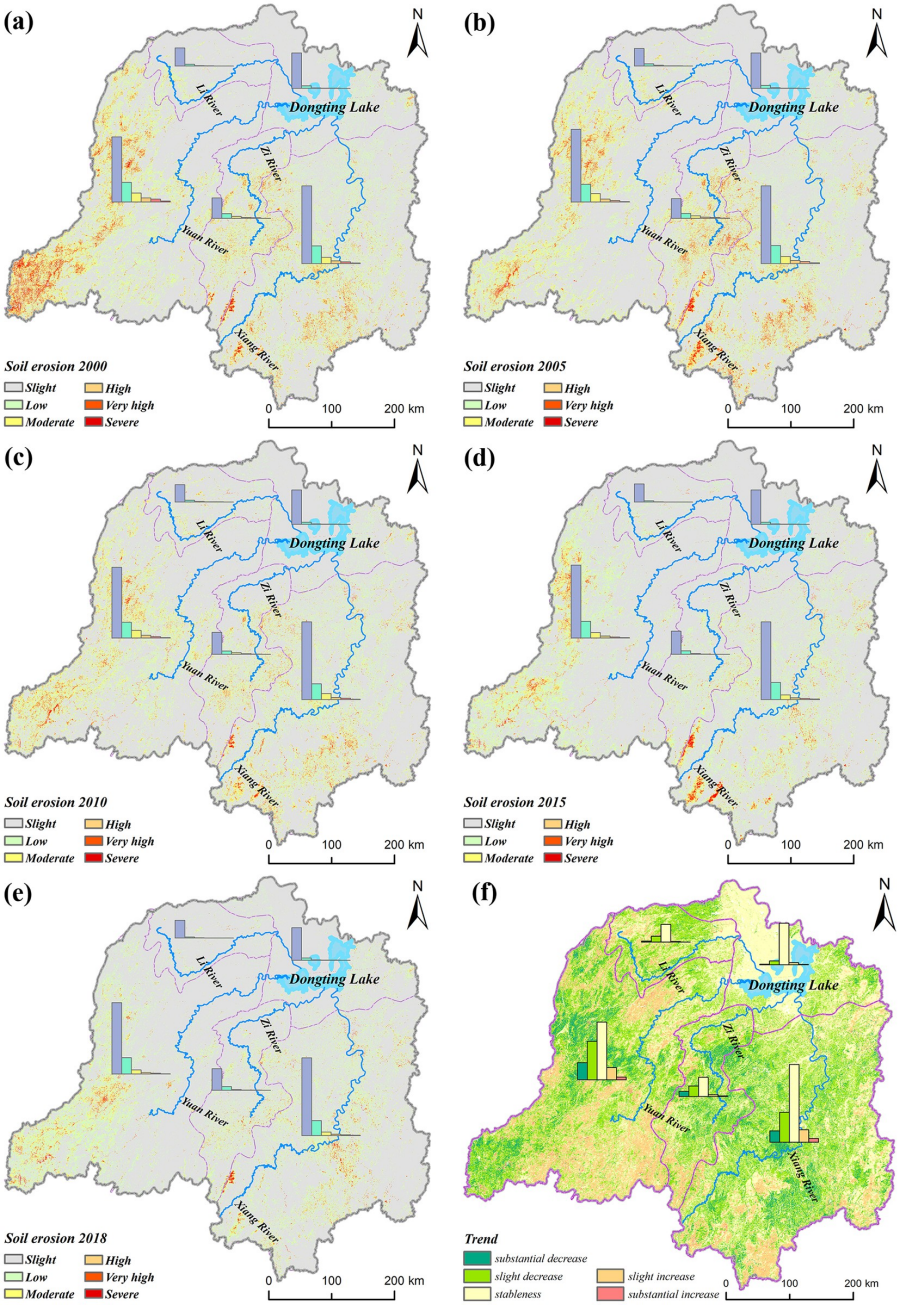
Spatial distribution of soil erosion in the study area from 2000 to 2018. (a) 2000, (b) 2005, (c) 2010, (d) 2015, and (e) 2018. (f) Trends of soil erosion in the study area from 2000 to 2018. The image was created using ARCGIS 10.2 software with the authors’ data.

Using 2018 as a cross section to analyze the spatial distribution characteristics of soil erosion (Fig 3e). The results show that the average erosion rate was 5.84 t∙hm^-2^ a^-1^. The soil loss area was 531 hm^2^, accounting for 20.20% of the study area. Slight erosion was the most common, whereas moderate, high, very high, and severe erosion accounted for 3.31%, 1.19%, 0.64%, and 0.26% of the total land area respectively (Fig 5). Fig 3e shows that most plain areas in the northern Dongting Lake Basin fell within the categories of slight erosion, and the soil loss primarily occurred in mountainous and hilly areas of the western and central-southern Dongting Lake Basin. In contrast, moderate and stronger erosion predominantly occurred in the grassland, garden land, and woodland areas in mountainous hills, which are characterized by steep slopes and low vegetation coverage. The most serious soil erosion was observed in the subbasins of the Yuan River Basin, with an average erosion modulus of 6.88 t∙hm^-2^ a^-1^. The soil loss area in the basin was 23,822 km^2^, accounting for 24.22% of the total area. The erosion modulus of the Xiang, Zi, and Li subwatersheds was 6.74, 5.88, and 3.46 t∙hm^-2^ a^-1^, respectively. The average erosion modulus of the area around Dongting Lake was 1.61 t∙hm^-2^ a^-1^. This area is characterized by flat terrain, and the main land use types are mainly water and paddy fields.

**Fig 4:**
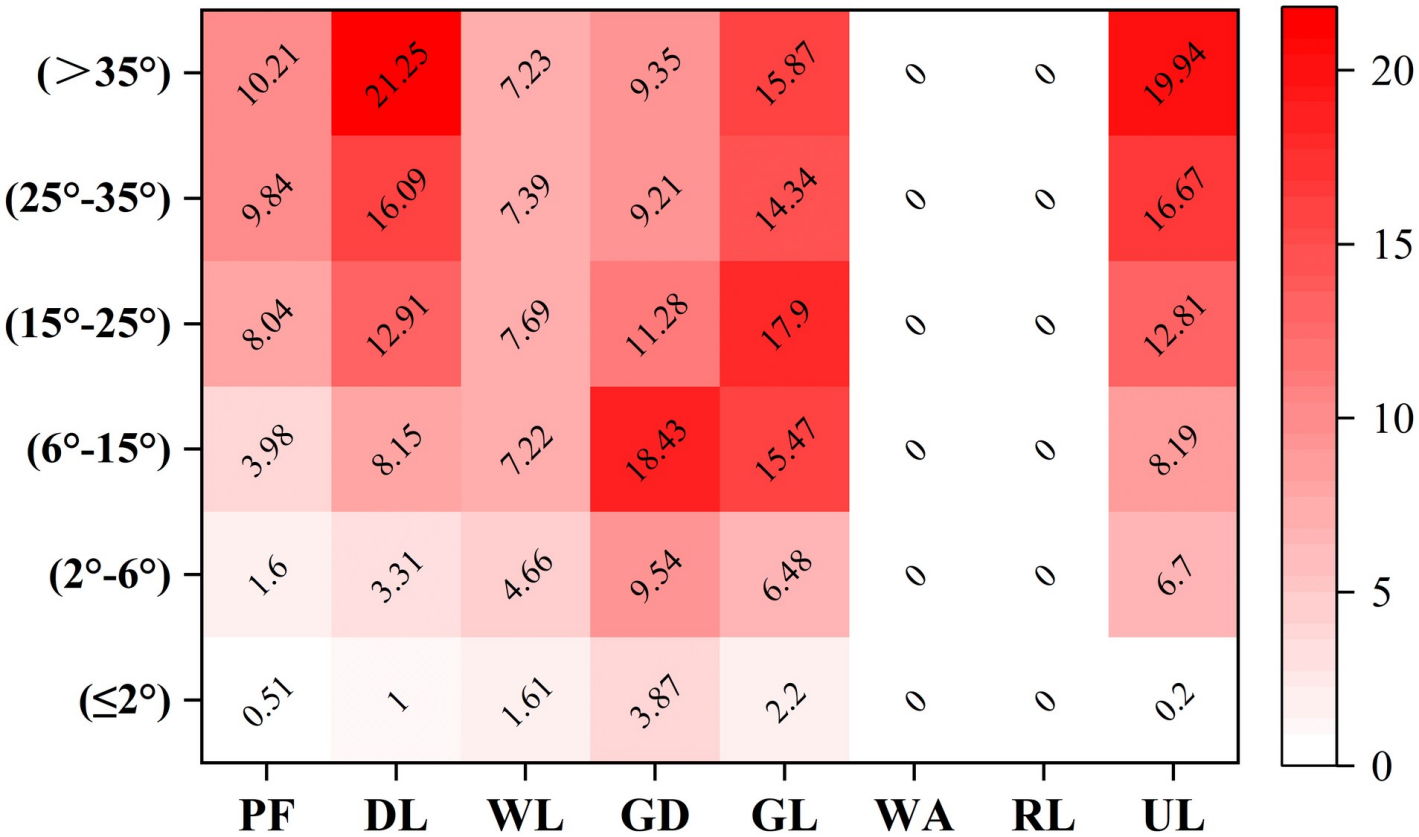
Soil erosion rates for each land use type under different slopes in the study area in 2018. PF—paddy field, DL—dry land, WL—woodland, GD—garden land, GL—grassland, WA—water area, RL—residential land, UL—unused land.

The land use and topography significantly affect the spatial distribution of soil erosion. The soil erosion of grassland was the most serious because the vegetation coverage of grassland was relatively low, and distributed in mountainous areas. The soil erosion rates of each land use type significantly differ under different slopes. The soil erosion rates of paddy fields, dry land, and unused land area increased with an increase in the slope. The soil erosion rates of woodland and garden land were divided by the slope of 15°. The soil erosion rate increases with the slope in the area with a slope of less than 15°, and the correlation between the soil erosion rate and the slope is not significant in the area with a slope of more than 15°. The soil erosion rate of the grassland area was divided by a slope of 25°. When the slope was less than 25°, the soil erosion rate increased with the slope. When the slope was greater than 25°, the correlation between the soil erosion rate and slope was insignificant (Fig 4). The average soil erosion rates of the paddy field, dry land, woodland, garden land, grassland, and unused land were 1.64, 5.89, 6.62, 12.34, 15.56, and 1.07 t∙hm^-2^ a^-1^ respectively.

### Dynamic changes of soil erosion

**Fig 5:**
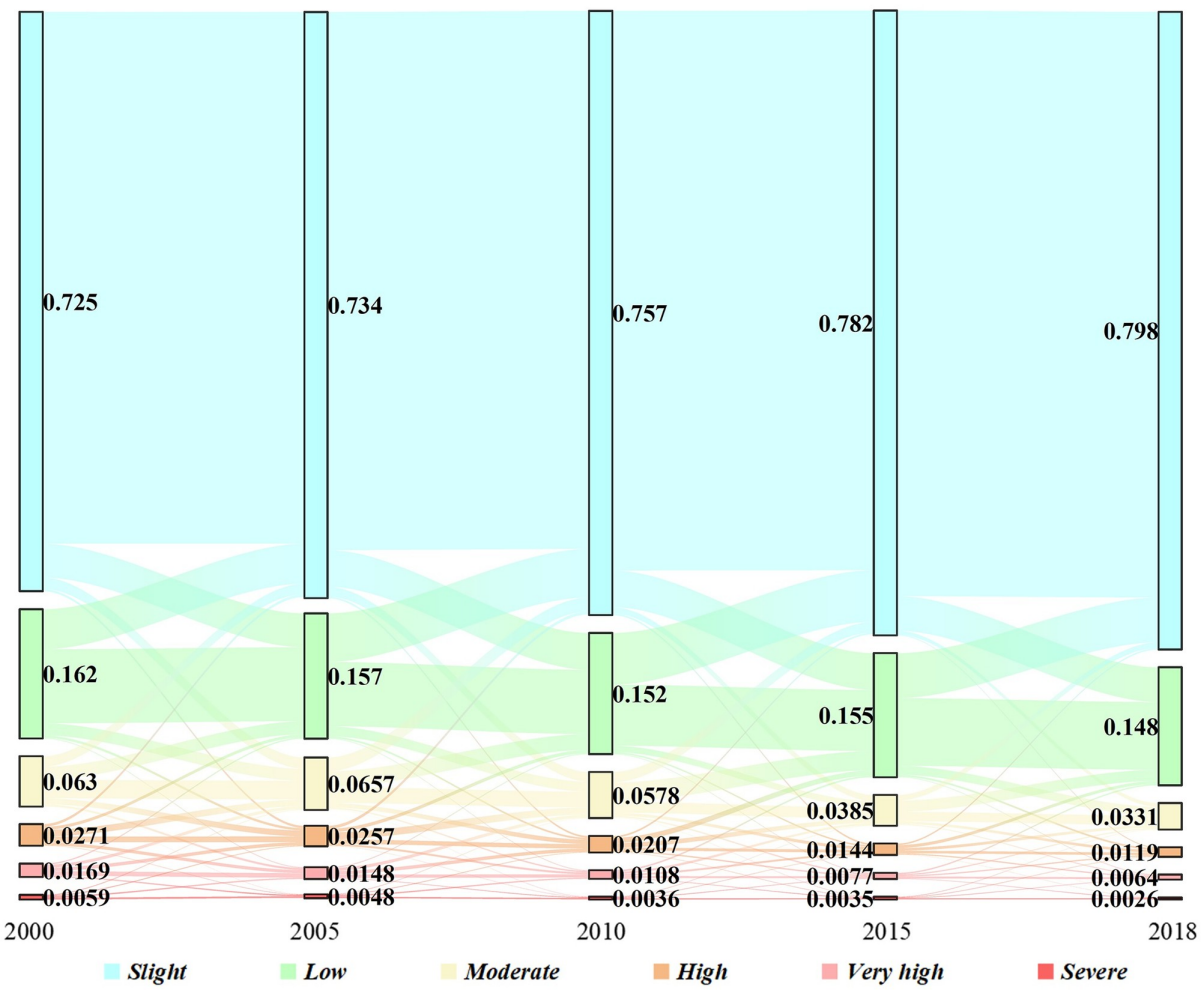
Change of the soil erosion intensity in the study area from 2000 to 2018.

The dynamic changes of the soil erosion from 2000 to 2018 were analyzed in this study. The average erosion rate in 2000, 2005, 2010, 2015, and 2018 was 10.63, 10.36, 8.98, 7.16, and 5.83 t∙hm^-2^ a^-1^, respectively, whereas the proportions of the soil loss area in these five years were 27.50%, 26.60%, 24.30%, 21.80%, and 20.20%, respectively. From 2000 to 2018, the average soil erosion rate and soil loss area decreased by 4.80 t∙hm^-2^ a^-1^ and 7.30% respectively. Generally, the area with low erosion continually decreased from 2000 to 2018. Except for a slight increase from 2000 to 2005, the moderately eroded area overall decreased. In addition, the areas with high, very high, and severe soil erosion showed a continuous decrease from 2000 to 2018 (Figs 3 and 5).

**Fig 6:**
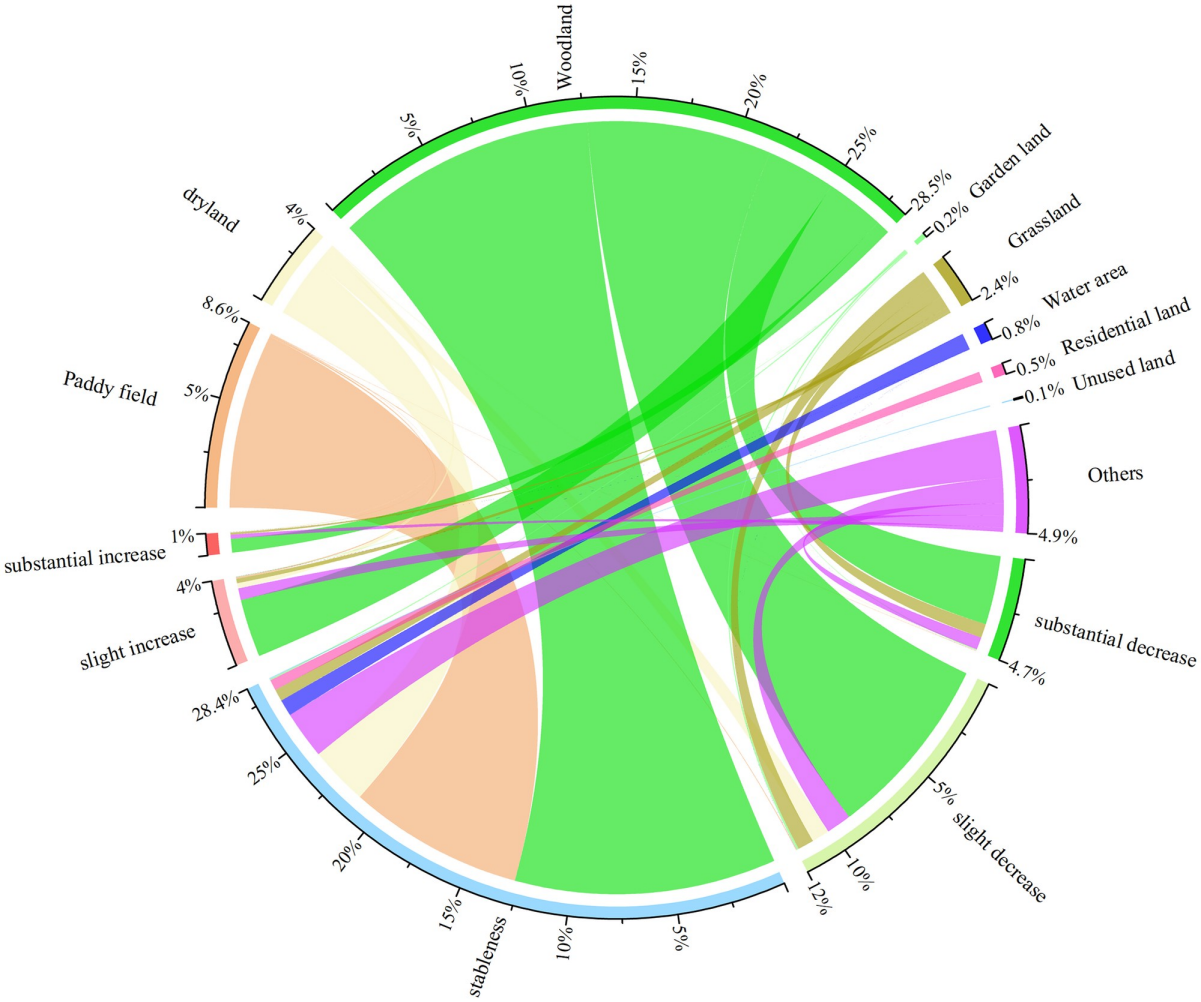
Statistics of the changes in the soil erosion based on different land use types in the study area from 2000 to 2018.

Changes in the soil erosion were analyzed using unary linear regression. The results show that 57.11% of the study area remained stable from 2000 to 2018. The stable area was mainly distributed in the Dongting Lake plain. Soil erosion decreased in 33.02% of the study area (including substantial decrease of 9.31%). It was mainly distributed in the upper reaches of the Yuan River and the middle reaches of the Zi River. In contrast, it increased in 9.87% of the area (including a substantial increase of 1.98%). The area with increased erosion was scattered in the middle reaches of the Xiang River, middle and upper reaches of the Yuan River, and several other areas (Fig 3f). In addition, the changes in the soil erosion depending on land use type were studied. Because the results of previous studies showed that changes in the land use type occurred in 9.81% of the entire study area, data on these areas were collected separately and categorized as *others*. Based on the statistics, the erosion modulus decreased in more than 50% of the garden land and grassland and in 43% of woodland (including a substantial decrease of more than 15% and 12%, respectively). Although the modulus increased in more than 10% of the garden land, woodland, and grassland, the substantial increase did not exceed 4%. Erosion insignificantly changed in paddy fields, dry land, and unused land. Note that the regional erosion modulus decreased (including a substantial decrease of 12%) in 37% in the areas with changes of land use types, whereas increases were observed in 16% of the region. (Including a substantial increase of 4%; Fig 6). In summary, the soil loss area decreased by 7.30% from 2010 to 2018, and the soil erosion decreased in 33.02% of the region. In all land use types, the areas with improved situations outweighed those with worsening situations and the improvements were the most remarkable in the grassland and garden land.

### Key factors affecting the spatial heterogeneity of soil erosion

**Fig 7:**
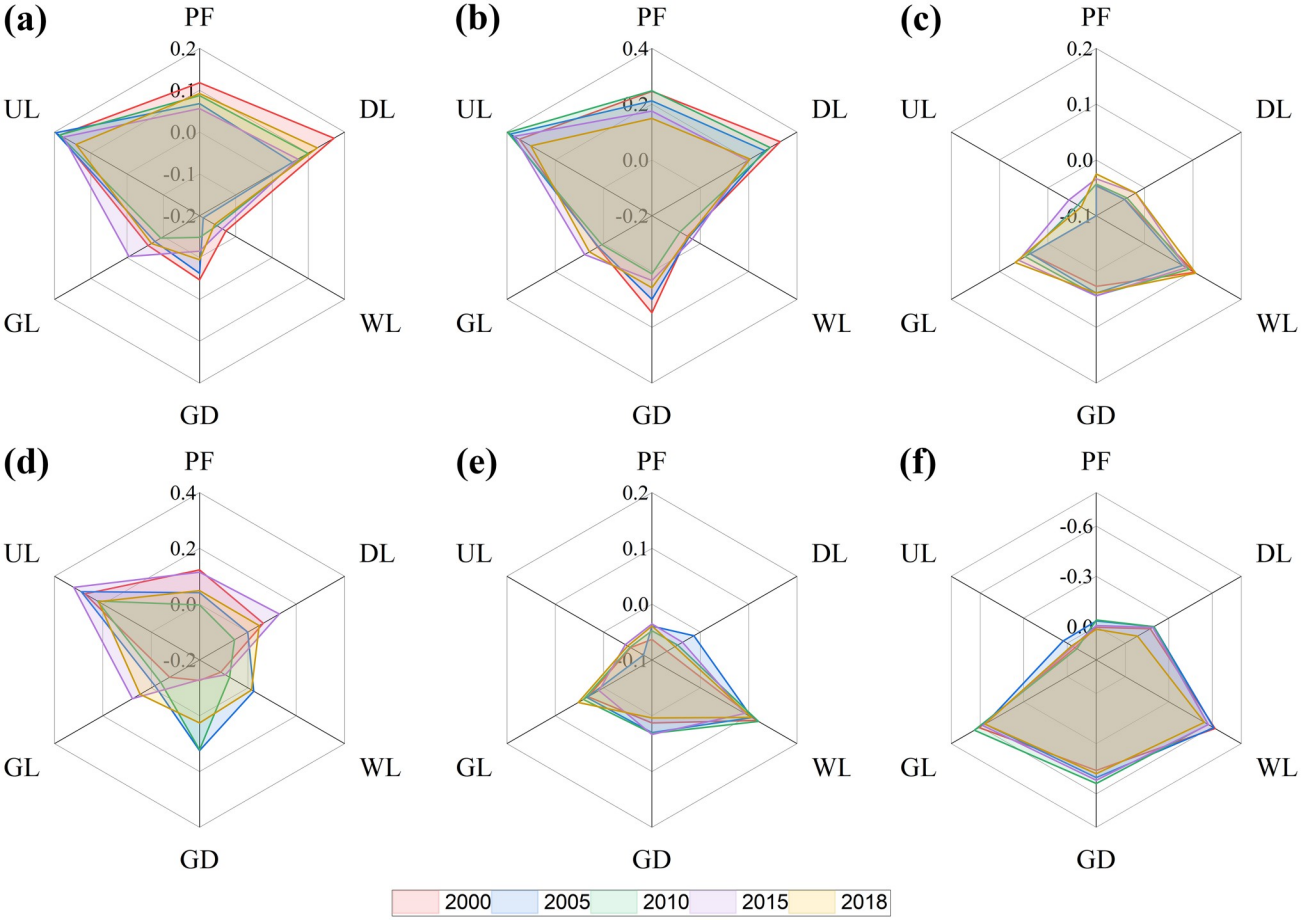
Effects of natural factors on soil erosion: (a) Elevation, (b) Slope, (c) Soil, (d) Rainfall, (e) Temperature, and (f) NDVI. PF—paddy field; DL—dry land; WL—woodland; GD—garden land; GL—grassland, and UL—unused land.

The spatial heterogeneity of soil erosion is affected by many factors, especially the terrain and climate [47, 48]. Based on previous studies, the elevation, slope steepness, soil, rainfall, temperature, and vegetation coverage (in this study, NDVI was used to represent the vegetation coverage) is the key factors affecting the heterogeneity of regional soil erosion [49–51]. We analyzed the correlations between these factors and the soil erosion rate for different land use types characterized by soil erosion (e.g., paddy fields, dry land, woodland, garden land, grassland, and unused land). The results are as follows. First, the correlation between the soil erosion rate and the elevation of paddy fields, woodlands, gardens, and grasslands is insignificant, whereas a positive correlation was observed between the soil erosion rate and the elevation of dry land and unused land, with a low correlation coefficient (Fig 7a). Second, the slope steepness of paddy fields, dry land, and unused land positively correlates with the soil erosion rate, with correlation coefficients exceeding 0.2 (Fig 7b). Third, the soil erosion rate positively correlates with the soil properties in woodland, gardens, and grassland, whereas the correlation between the soil erosion rate and soil properties in unused land is negative. In both cases, the correlation coefficient is relatively low (Fig 7c). Fourth, the soil erosion rate and rainfall in dry land, woodland, garden land, and grassland, whereas a positive correlation was observed in unused land, with a correlation coefficient of ~0.2 (Fig 7d). Fifth, the temperature and soil erosion rate in woodland, garden land, and grassland positively correlate, with a low correlation coefficient. The temperature in paddy fields, dry land, and unused land did not correlate with the soil erosion rate (Fig 7e). Sixth, the NDVI in woodland, gardens, and grassland negatively correlates with the soil erosion rate. All the correlation coefficients were larger than 0.5. The NDVI in paddy fields, dry land, and unused land did not correlate with the soil erosion rate (Fig 7f). In summary, the slope steepness is the main factor affecting the intensity of soil erosion in paddy fields, dry land, and unused land and the vegetation coverage is the main factor affecting the intensity of soil erosion in woodlands, gardens, and grasslands.

### Contribution of soil erosion changes

**Fig 8:**
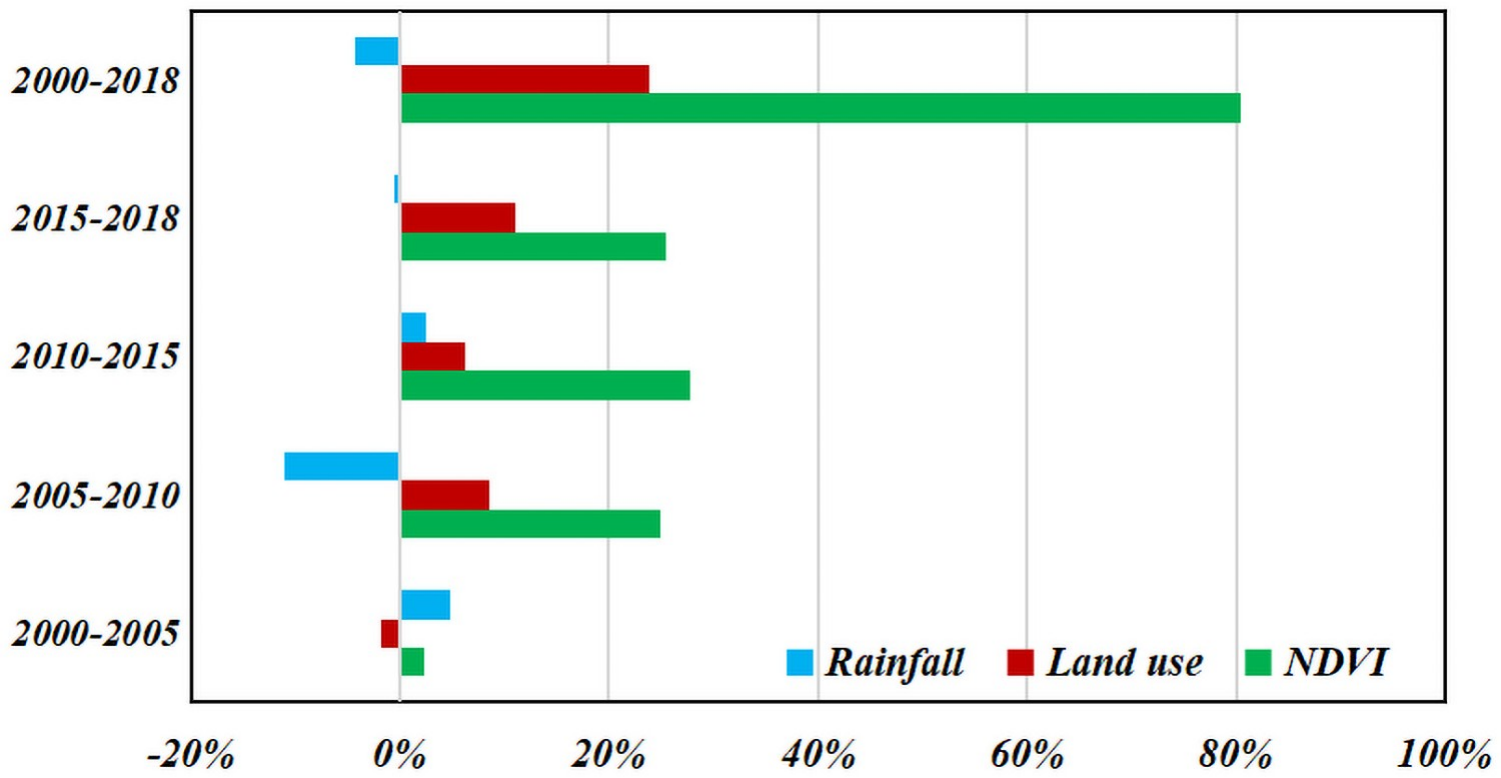
Relative contributions of rainfall, land use, and NDVI to soil erosion changes during four periods.

The relative contributions of climate change and human activities to soil erosion changes were determined to use a sensitivity experiment in this study. From 2000 to 2018, the increase in the vegetation coverage played a major role in alleviating soil erosion, with a contribution rate of 80.40%. The contribution rate of land use transfer to soil erosion improvement was 23.92%. In contrast, increases in the rainfall levels and intensity led to increased soil erosion (increased rainfall has a negative impact on soil erosion, with a contribution rate of -4.32%). Human activities, such as the transfer of land use and changes in the vegetation coverage, significantly contributed to the alleviation of soil erosion in the Dongting Lake Basin, whereas changes in rainfall as a representative of climate change slightly intensified the soil erosion in the study area. The relative contribution rate varied in at different periods. Rainfall changes played a positive role in alleviating soil erosion in 2000–2005 and 2010–2015; in contrast, they played a negative role in 2005–2010 and 2015–2018. Land use transfer played a positive role in alleviating soil erosion in 2005–2018, but it played a negative role in 2000–2005. Changes in vegetation coverage had positive effects on soil erosion, compared with other periods, the contribution rate in the first period (2000–2005) was low (Fig 8).

### Effects of land use transformation on soil erosion

**Fig 9:**
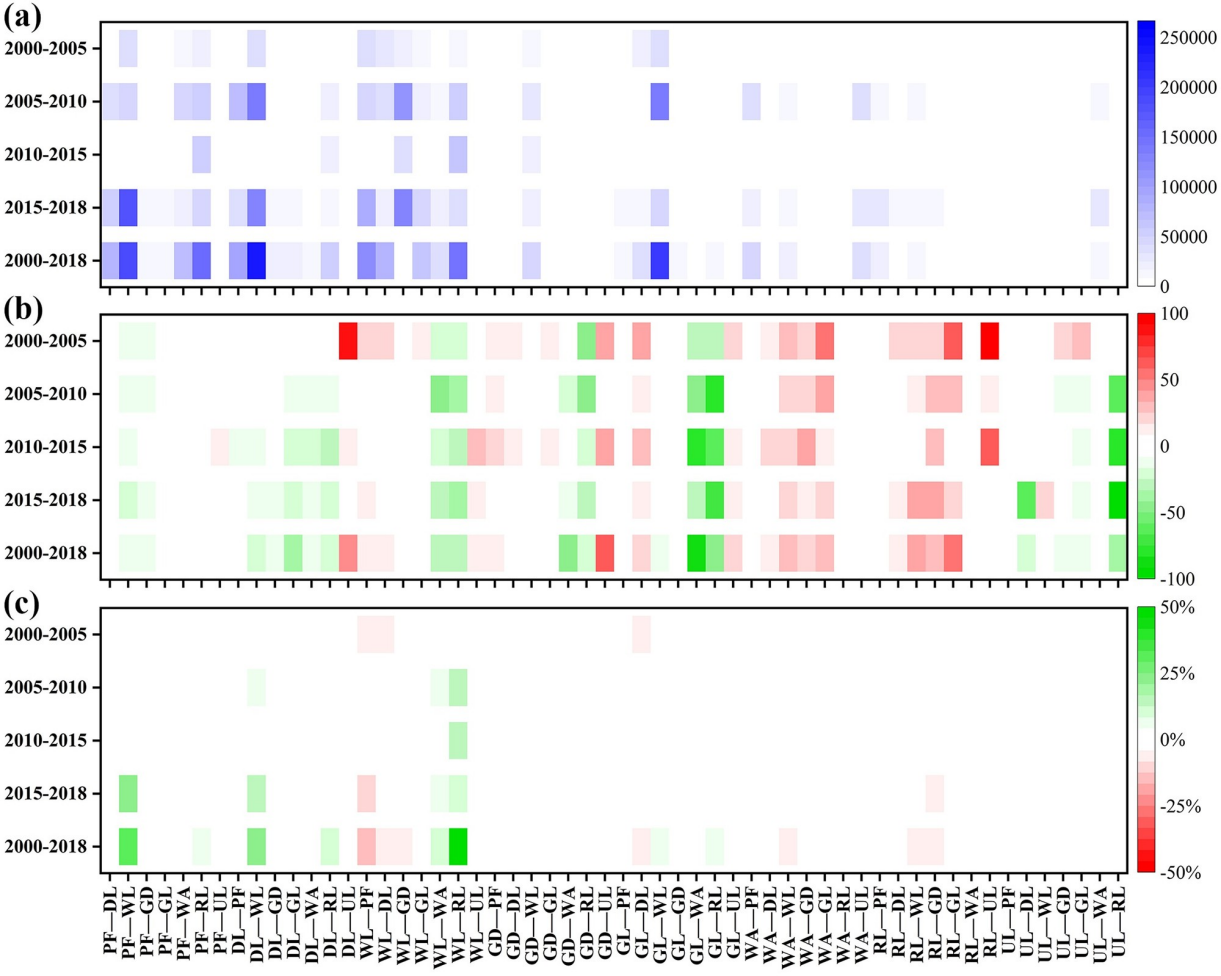
(a) Changes in each land use type area (hm^2^) during four periods. (b) Changes in the soil erosion rate (MJ mm hm^-2^ h^-1^ a^-1^) for each land use transformation during four periods. (c) Contributions (percentage) of each land use transformation to soil erosion changes during four periods. PF—paddy field, DL—dry land, WL—woodland, GD—garden land, GL—grassland, WA—water area, RL—residential land, and UL—unused land.

From 2000 to 2018, changes in the land use types occurred in 9.81% of the total area. Fig 9a shows the direction of the transfer and the area undergoing changes. The main transfer directions are as follows: paddy fields to woodland, water areas, and construction land, dry land to woodland and construction land, woodland to garden land, construction land, and partly to paddy field and dry land; and grassland to woodland. As a result of the transfer, the area of paddy fields, dry land, woodland, and grassland decreased by 130,000, 180,000, 140,000, and 190,000 hm^2^ respectively, whereas the areas of construction land, garden land, water area, and unused land increased by 330,000, 270,000, 30,000, and 10,000 hm^2^ respectively.

The changes in the soil erosion rate of each land use type from 2000 to 2018, which were affected by the topography, are shown in Fig 9b. In regions with a slope below 6°, the erosion intensity did not respond to the transfer of each land use type. In regions with steep slopes, the transfer from paddy fields and dry land to woodland or gardens increased the vegetation coverage and further reduced the soil erosion rate. The transfer from paddy fields, dry land, and woodland to construction land led to the hardening of the ground, resulting in a decreased soil erosion rate. In contrast, the transfer in the opposite direction increased soil erosion, which might have been caused by timber cutting from 2000 to 2005.

Fig 9c shows the contribution of each type of land use transfer to the change in soil erosion intensity from 2000 to 2018. The transfer from woodland to construction land contributed to up to 46% of the soil erosion improvement, whereas the transfer from paddy fields and dry land to woodland contributed to 30% and 23%, respectively, and the transfer from paddy fields and dry land to construction land led to an improvement of ~7% and 9%, respectively. In contrast, the transfer from woodland to paddy fields, dry land, and garden land intensified the soil erosion, with contribution rates of -15%, -7%, and -5%, respectively. The contribution rates differed in different periods. From 2000 to 2005, land use changes aggravated the soil erosion, with a contribution rate of -6%, from 2005 to 2010, 2010 to 2015, and 2015 to 2018, soil erosion improved due to land use changes, with contribution rates of 36%, 25%, and 45%, respectively.

### Effects of vegetation cover changes on soil erosion

**Fig 10:**
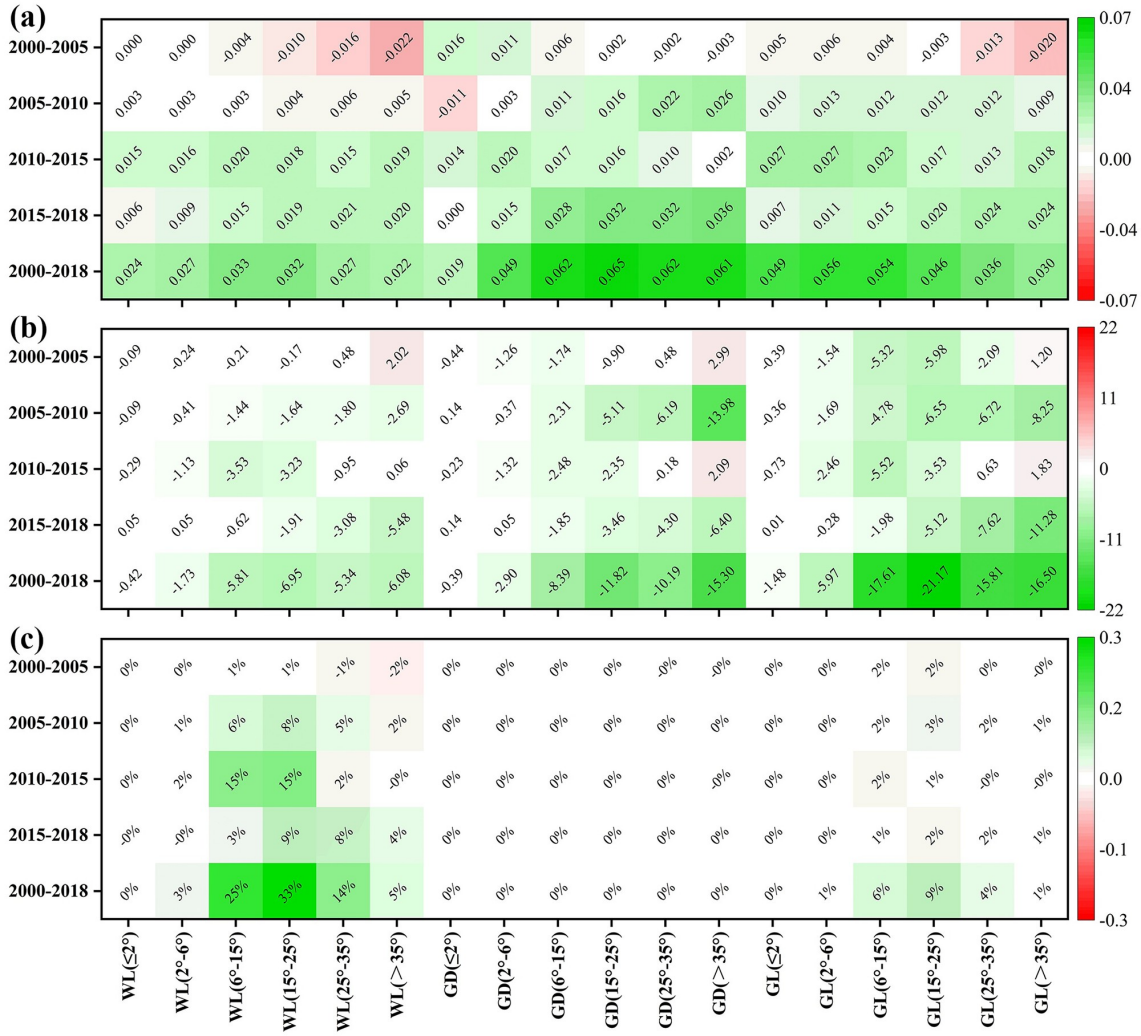
(a) Changes in the NDVI of each land use type during four periods. (b) Changes in soil erosion rates (MJ mm hm^-2^ h^-1^ a^-1^) of each land use type during four periods. (c) Contributions (%) of the changes of NDVI of each land use type to soil erosion changes during four periods. WL—woodland, GD—garden land, and GL—grassland.

Based on the analyses in Sections 3.2 and 3.3, it can be concluded that the soil erosion rates of forest land, garden land, and grassland significantly changed and vegetation coverage was the dominant factor affecting the soil erosion rates in these areas. The vegetation coverage in the Dongting Lake Basin significantly changed from 2000 to 2018. Although both increases and decreases in the coverage rates occurred in different terrains during the first five years, an overall increase was observed from 2005 to 2018. The restoration of the vegetation cover in the garden land was the most significant among all land use types, followed by grassland and woodland. Fig 10a shows the changes in the vegetation coverage rates in garden land, woodland, and grassland for different slopes. Vegetation restoration was relatively significant when the slope was larger than 15° in garden land and between 6° to 25° in woodland. Vegetation restoration was comparatively significant when the slope less steep.

Fig 10b shows the dynamic changes in the soil erosion rate for each land use type and different slopes. Based on the chart, the improvement of the soil erosion in the grassland area was the most significant. The soil erosion rate of the grassland responded more positively to changes in the vegetation coverage, especially in areas with a slope above 15°. The improvement of soil erosion was more significant in woodland and garden land with slopes above 6°. The level of the responses of the soil erosion rate to changes in the vegetation coverage increased as the slope increased.

The contributions of vegetation restoration to the improvement of soil erosion were assessed based on the changes in the soil erosion rates and the areas of the three land use types (Fig 10c). Vegetation restoration contributed the most to the improvement in woodland areas. The contribution rate reached 33% at a slope ranging from 15–25°. The contribution rate of the grassland area was 20%; it was 9% in areas with a slope between 15–25°. The contribution of vegetation restoration to the improvement of soil erosion significantly varied in different periods. From 2000 to 2005, the lowest contribution rate was 4%. In 2005–2010, 2010–2015, and 2015–2018, the rates insignificantly differed (28%, 38%, and 30%, respectively).

## Discussion

### Verification of the results

**Fig 11:**
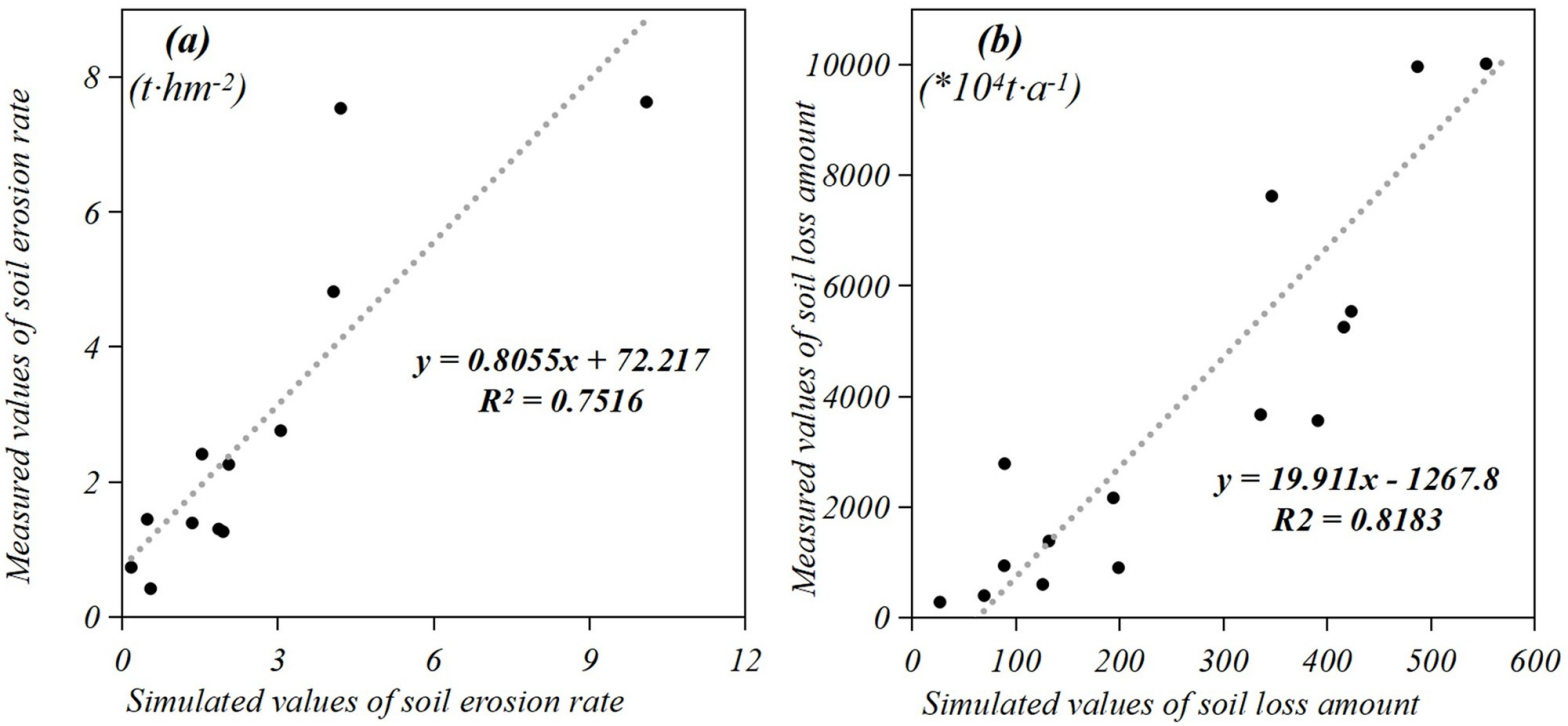
Verification of the soil erosion rate and soil loss amount.

Because RUSLE is an empirical model, it may be challenging to verify the results within the watershed. In this study, the results of the RUSLE simulations were verified with data measured using existing methods [8, 52]. The measured data regarding the local soil erosion modulus and sediment yield were obtained from four surface engineering monitoring stations and four hydrological monitoring stations. The four engineering monitoring stations included Qiubo, Lianhe, Qingtang, and Ximixiang Station (locations are shown in Fig 1). The simulated soil erosion values were compared with the data measured at these four stations in 2010, 2015, and 2018. Based on the results, the correlation coefficient was R^2^ = 0.75 (Fig 11a), indicating a strong correlation between the two variables. The four hydrological monitoring stations included Hengyang (Xiang River), Taojiang (Zi River), Taoyuan (Yuan River), and Shimen stations (Li River; the locations are shown in Fig 1). The simulated soil loss was compared with the sediment runoff in the watershed measured in 2005, 2010, 2015, and 2018. The correlation coefficient was determined to be 0.82 (Fig 11b), indicating a strong correlation between the two variables.

For further verification, the soil erosion rate in this study was compared with that determined in other studies. In this study, the annual soil erosion rate ranged from 5.83–10.63 t∙hm^-2^ a^-1^. By adopting sampling methods, Liu estimated that the average soil erosion rate in China in 2011 was 5 t∙hm^-2^ a^-1^ [53]. Qian et al. used the revised RUSLE model and reported that the soil erosion rate in southern China in 2015 was 24.73 t∙hm^-2^ a^-1^ [17]. Chen et al. used the RUSLE and estimated the soil erosion rate in the Hunan Province in 2010 to be 5.58 t∙hm^-2^ a^-1^ [37]. Differences in the data and methods may explain the differences in specific values. Based on the two rounds of verification, it can be concluded that the accuracy of the simulated results in this study meets the evaluation and application requirements.

### Factors influencing the spatial heterogeneity of soil erosion

The capacity of soil conservation of different land use types differs [53]. Each land use type affects the soil erosion by influencing the underground biomass and surface vegetation cover with different intensities [54, 55]. The erosion rate in woodland is lower than that in garden land and grassland because the vegetation above the ground in woodland is denser and the root system underground is more developed. The interception of raindrops by vegetation decreases the velocity of raindrops and prevents them from directly impacting the soil surface particles. Furthermore, denser vegetation slows down the overland water flow and the root systems of trees and shrubs plays an important role in decreasing the runoff by improving the soil characteristics [49]. The topography can accelerate or slow down the soil loss, especially in the hilly areas of southern China. In steeply sloped areas, dry land and bare soil increase the susceptibility of soil to erosion, because the low vegetation coverage causes the exposure of soil particles to raindrops, leading to their detachment. In plain areas, almost no erosion was observed, and the degree of erosion and intensity of land use insignificantly correlate [47]. The erosion rates determined in paddy fields in this study were low because the paddy fields were mostly distributed in the plain areas of the Dongting Lake Basin. With respect to paddy fields in mountainous areas, terraces play important roles in soil and water conservation [56]. Grassland, garden land, and woodland are mainly distributed in steeply sloped areas, whereas dry land, paddy fields, and unused land are common in plain areas. The vegetation coverage of forest land is higher than that of garden land and grassland. Based on previous studies, the topography and vegetation coverage are two major factors accounting for the differences in the soil erosion rates of different land use types [53, 57].

The driving factors of soil erosion differ depending on the land use type. The slope steepness is the key factor affecting the intensity of soil erosion in the dry land, paddy fields, and unused land, whereas the vegetation coverage is the key factor affecting the intensity of soil erosion in woodland, garden land, and grassland. The main body of the Dongting Lake Basin is located in the red soil hilly area of southern China, which are under the influence of mesoscale subtropical monsoon climate. The spatial differences in the temperature and precipitation are insignificant and the differences in soil characteristics are small. However, the topography and vegetation coverage of different land use types notably differ [58]. The spatial heterogeneity of the vegetation coverage in paddy fields and dry land is small and the slope is the main factor affecting the soil erosion rate in dry land [59]. Soil erosion occurs more frequently in bare soil in unused land because the soil in bare land with large slopes is more susceptible to rainfall and might be transported away without the protection from vegetation [60]. Agriculture, forestry, and animal husbandry are frequent activities in areas with gentle slopes, which might cause serious water and soil loss in woodland, garden land, and grassland. Therefore, the vegetation coverage plays a vital role in soil conservation in these areas [61, 62].

### Driving factors of the spatiotemporal dynamics of soil erosion

Factors, such as the land use and climate, drive changes in soil erosion [63]. Human activities represented by land use types and vegetation coverage significantly contributed to the alleviation of soil erosion in the Dongting Lake Basin, whereas climate change represented by rainfall slightly aggravated soil erosion in the study area. Rapid economic growth and urbanization have promoted the transfer of woodland, paddy fields, and dry land to construction land in the suburbs of the Dongting Lake Basin from 2000 to 2018 [36]. This transfer of land led to the hardening of the ground, resulting in a decreased soil erosion rate. Based on the Grain for Green Program implemented by the Chinese government, paddy fields, and dryland to forest land with slopes greater than 25° and high soil erosion rates have been transferred to forest land, the vegetation coverage has increased, while effectively alleviating the problem of soil erosion [43]. The Natural Forest Protection Program, another program initiated in China, led to the reduction of agricultural, forestry, and animal husbandry activities and the interference of human activities in the protected area, thus promoting the restoration of vegetation in woodlands, gardens, and grassland [64]. Rainfall is a direct factor affecting the soil erosion. It has been projected that increases in the precipitation levels and intensity due to climate change will lead to increased erosion [65]. From 2000 to 2018, the rainfall increased in different land use types and higher rainfall amounts intensified the soil erosion. The effect of the climate on soil erosion is more complex [48]. Climate change affects the erosivity by influencing erosion through concomitant changes in plant community dynamics and disturbance regimes such as fire, drought, and desertification [66, 67].

### Implications and limitations of the study

In this study, the differences in the soil erosion of various land use types in the Dongting Lake Basin were determined and changes in the soil erosion were analyzed. The dominant factors affecting soil erosion in each land use type were identified and the relative contributions of human activities and climate change to soil erosion changes were determined. We explored the effects of different land use types and vegetation cover restoration on soil erosion. The results of this study promote the soil conservation in the Dongting Lake Basin. However, this study has several limitations. It is imperfect for land use types and vegetation coverage to replace human activities. The quantification of human activities is a current research hotspot. Only the direct contribution of climate change to soil erosion was assessed in this study. In future studies, the contribution of climate change to soil erosion should be assessed while discriminating between direct and indirect impacts.

## Conclusions

In this study, the characteristics and driving mechanisms of soil erosion in the Dongting Lake Basin were explored. The following conclusions can be drawn: (1) From 2000 to 2018, both the soil erosion rate and area of soil loss in the Dongting Lake Basin decreased. Human activities represented by land use types and vegetation coverage significantly contributed to the alleviation of the soil erosion in the Dongting Lake Basin, whereas climate change represented by rainfall slightly aggravated the soil erosion in the study area. (2) The quantification of the effects of different factors in each land use type on soil erosion showed that the slope steepness is the key factor affecting the intensity of soil erosion in dry land, paddy fields, and unused land. Whereas the vegetation coverage is the key factor affecting the intensity of soil erosion in woodland, garden land, and grassland. (3) The transfer from paddy fields and dry land to woodland, the transfer of woodland, paddy fields, and dry land to construction land, and the restoration of woodland vegetation significantly contribute to the alleviation of soil erosion in the study area. At the same time, the restoration of grassland vegetation has the largest effect on the alleviation of soil erosion. Hence, when preventing and controlling soil erosion, more attention should be paid to land management and the restoration of vegetation coverage.

## Supporting information

S1 Fig Land use patterns changes in the study area from 2000 to 2018. (DOCX)

S2 Fig NDVI changes in the study area from 2000 to 2018. (DOCX)

S1 Table Land use types area and proportion in the study area from 2000 to 2018. (DOCX)
