# Supplementary material for: Assessment of soil erosion in the Dongting Lake Basin, China: Patterns, drivers, and implications
Source: PLoS One. 2021 Dec 31;16(12):e0261842. doi: 10.1371/journal.pone.0261842 (PMC8719766; doi:10.1371/journal.pone.0261842)
Supplement: S2 Fig — (DOCX) [file pone.0261842.s002.docx]

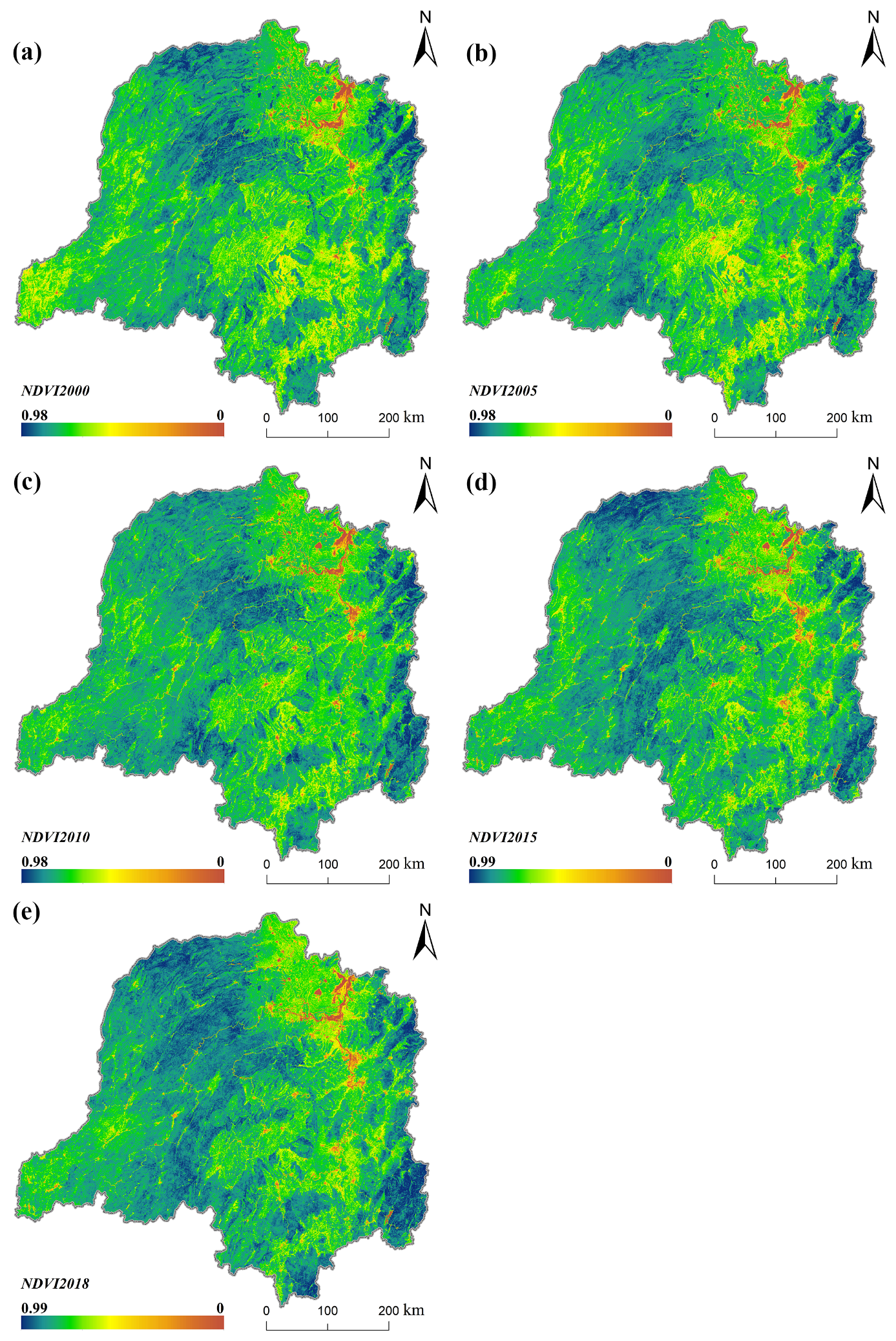


**S2 Fig. NDVI changes in the study area from 2000 to 2018. (a)2000; (b)2005; (c)2010; (d)2015; (e)2018;** The image was created using ARCGIS 10.2 software with the authors' data.
