# Supplementary material for: Assessment of soil erosion in the Dongting Lake Basin, China: Patterns, drivers, and implications
Source: PLoS One. 2021 Dec 31;16(12):e0261842. doi: 10.1371/journal.pone.0261842 (PMC8719766; doi:10.1371/journal.pone.0261842)
Supplement: S1 Table — (DOCX) [file pone.0261842.s003.docx]

**S1 Table.** Land use types area and proportion in the study area from 2000 to 2018.

| **Landuse** | **2000** | **2005** | **2010** | **2015** | **2018** |
| --- | --- | --- | --- | --- | --- |
| **Paddy field** | 51159.70 | 49541.27 | 50633.45 | 50061.98 | 49813.14 |
|  | 19.47% | 18.85% | 19.27% | 19.05% | 18.95% |
| **Dryland** | 25842.80 | 26003.78 | 24280.25 | 24121.38 | 24068.73 |
|  | 9.83% | 9.89% | 9.24% | 9.18% | 9.16% |
| **Woodland** | 159324.09 | 160296.91 | 160293.11 | 159377.71 | 157927.74 |
|  | 60.63% | 61.00% | 60.99% | 60.65% | 60.09% |
| **Garden land** | 1808.56 | 1945.29 | 2888.92 | 3122.94 | 4476.47 |
|  | 0.69% | 0.74% | 1.10% | 1.19% | 1.70% |
| **Grassland** | 15372.99 | 15007.09 | 13633.24 | 13506.03 | 13461.99 |
|  | 5.85% | 5.71% | 5.19% | 5.14% | 5.12% |
| **Water area** | 5592.79 | 5807.91 | 5665.31 | 5731.94 | 5913.24 |
|  | 2.13% | 2.21% | 2.16% | 2.18% | 2.25% |
| **Residential land** | 3247.84 | 3680.59 | 4733.39 | 6191.51 | 6555.93 |
|  | 1.24% | 1.40% | 1.80% | 2.36% | 2.49% |
| **Unused land** | 451.23 | 517.17 | 672.32 | 686.51 | 582.76 |
|  | 0.17% | 0.20% | 0.26% | 0.26% | 0.22% |
